# Microarteriovenous Fistulas Causing Refractory Skin Ulcers: Feasibility and Safety of Transcatheter Embolization

**DOI:** 10.1007/s00270-025-04234-0

**Published:** 2025-10-19

**Authors:** Sota Oguro, Akira Endo, Hiromitsu Tannai, Hideki Ota, Tomomi Sato, Tomoki Sato, Hiroki Kamada, Yosuke Miyachi, Masato Ito, Hiroaki Furukawa, Yumi Kambayashi, Yoshihide Asano, Fukashi Serizawa, Daijirou Akamatsu, Kei Takase, Shigeki Imai

**Affiliations:** 1 Department of Diagnostic Radiology, Tohoku University Hospital, Sendai, Miyagi 980-8574 Japan; 2 Department of Dermatology, Tohoku University Graduate School of Medicine, Sendai, Miyagi 980-8574 Japan; 3 Department of Surgery, Tohoku University Hospital, Sendai, Miyagi 980-8574 Japan; 4 Department of Vascular and Interventional Radiology, Southern Tohoku General Hospital, Fukushima, 963-8052 Japan

**Keywords:** Micro-arteriovenous fistula, Transcatheter arterial embolization, Imipenem/cilastatin sodium, Refractory skin ulcer, Lower Iimb

## Abstract

**Purpose:**

Microarteriovenous fistulas (m-AVFs) have been proposed as a potential cause of lower limb ulcers that are refractory to standard therapies. This study evaluated the feasibility and safety of transcatheter arterial embolization (TAE) using imipenem/cilastatin sodium (IPM/CS) in patients with m-AVF-related refractory skin ulcers.

**Methods:**

This retrospective study included 17 patients with lower limb refractory skin ulcers treated with TAE from 2013 to 2023. M-AVFs were diagnosed via Doppler and confirmed by angiography. Embolizations were done under local anesthesia through femoral artery access, using imipenem/cilastatin mixed with contrast, injected until flow stagnation or a 0.5 g max.

Technical success was defined as the elimination of early venous shunting on angiography. Clinical success was defined as a ≥ 50% reduction in ulcer size. Repeated TAE procedures were performed at 1–2-month intervals when clinical improvement was deemed insufficient, primarily based on physician judgment regarding ulcer size and pain. While no strict quantitative threshold was used, the decision to proceed with additional sessions beyond three was based on discussion between the patient and physician. The outcome evaluation included assessments of ulceration and pain severity.

**Results:**

We performed 41 embolizations on 17 patients, with repeat sessions every 1–2 months if needed. Technical success was achieved in all cases (100%). No major complications occurred; minor pain was reported in ~ 20% of cases. Over an average 11.5-month follow-up, clinical success was observed in 88% of patients.

**Conclusion:**

TAE with IPM/CS is a feasible and safe treatment option for refractory lower limb skin ulcers caused by m-AVFs.

**Graphical abstract:**

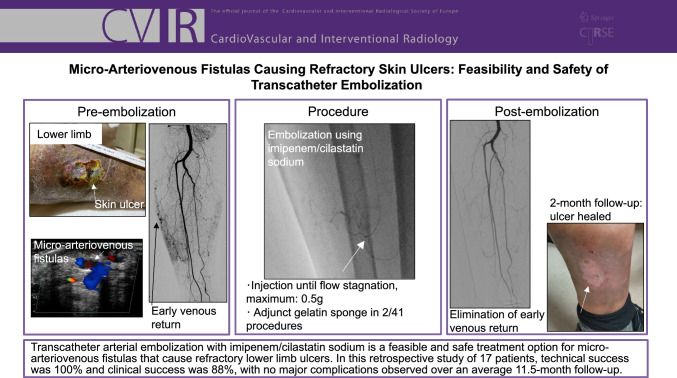

## Introduction

Chronic lower limb ulcers are a debilitating condition that often persists despite standard therapies such as compression, limb elevation, and wound care. While most cases are attributed to well-known etiologies, such as chronic venous insufficiency, lymphedema, or arterial disease, a subset of ulcers remains refractory even after prolonged treatment [1–6]. Emerging case reports suggest that microarteriovenous fistulas (m-AVFs) are an underrecognized cause of such refractory ulcers [7, 8]. These small shunts allow high-pressure arterial flow to bypass the capillary network directly into the venous system, causing venous hypertension, tissue congestion, and impaired microcirculatory exchange, which may impede healing [7, 9]. As a result, management of m-AVFs remains challenging. Surgical ligation is often impractical because of the small caliber and diffuse distribution of fistulas, and recanalization or collateral formation frequently leads to recurrence. Sclerotherapy has also been ineffective for high-flow lesions such as m-AVFs [9, 10]. Although transcatheter arterial embolization (TAE) is a minimally invasive alternative, concerns about ischemic complications and worsening of ulcers with permanent embolic agents have limited its application.

Imipenem/cilastatin sodium (IPM/CS) has recently been proposed as a novel temporary embolic agent that may overcome these limitations. When mixed with contrast, IPM/CS forms fine microparticles that selectively occlude abnormal microvessels and dissolve rapidly in vivo [11–13]. Preclinical studies have demonstrated its ability to occlude pathological shunting without compromising normal tissue perfusion [14].

We hypothesized that selective embolization of m-AVFs using IPM/CS could relieve venous hypertension and promote healing in otherwise refractory lower limb ulcers.

## Materials and Methods

### Patient Selection

This retrospective study enrolled patients from Tohoku University Hospital and Southern Tohoku General Hospital, who underwent TAE for treatment-resistant lower limb refractory skin ulcers and associated symptoms between June 2013 and May 2023. This study was approved by the Institutional Ethics Committee (approval number: 2022–1-560), which granted a waiver of informed consent for the retrospective analysis. Nevertheless, written informed consent for the procedures was obtained from all patients before they participated in the study. The diagnosis of m-AVFs was based on Doppler ultrasonography findings, which showed continuous arterialized waveforms in superficial veins with communication from deep arteries. Angiography further confirmed m-AVFs by demonstrating early venous return without evidence of a nidus or venous pouch [11]. All 17 patients underwent Doppler ultrasonography as part of the diagnostic workup before angiography. Patients qualified if they had failed at least 3 months of conservative treatment, such as compression or wound care. Exclusion criteria included deep vein thrombosis, hypoalbuminemia, lymphatic obstruction, myxedema, malignancies, varicose veins, or inability to comply with conservative measures. Ongoing infection did not disqualify participation.

### Endovascular Procedure

All TAE procedures were performed under local anesthesia. After placing a 4-French catheter into the superficial femoral artery (SFA), digital subtraction angiography (DSA) of the SFA was routinely performed to assess early venous return and collateral circulation. A 0.016-inch microguidewire (Asahi Meister, ASAHI INTECC, Japan) and 1.7-French microcatheter (Veloute, ASAHI INTECC, Japan) were coaxially advanced into the feeding arteries of the m-AVF. The m-AVFs exhibited faint, ill-defined enhancement with early venous return on angiography. IPM/CS (0.5 g) mixed with 5 mL of iodinated contrast creates 10–70 μm microparticles, and this mixture was injected into each vessel in 0.2-mL increments until flow stagnation or a maximum of 5 mL [12]. In cases where residual early venous shunting persisted after IPM/CS injection, additional embolization with gelatin sponge (Spongel®, Astellas, Tokyo, Japan) was performed.

### Outcome Evaluation

Technical success was defined as the complete elimination of early venous reflux on angiography, regardless of whether additional embolic agents (e.g., gelatin sponge) were required to achieve this endpoint. Adverse events were classified under the Common Terminology Criteria for Adverse Events (CTCAE) v5.0 [15]. Pain severity, as a secondary outcome, was measured on a 0–10 numerical rating scale (NRS). Dermatologists evaluated ulcers at one and three months, categorizing changes as improved (≥ 50% reduction), partially improved (≥ 10%– < 50%), unchanged (< 10%), or worsened (increase). Clinical success was defined as a ≥ 50% reduction in ulcer size. Repeated TAE procedures were performed at 1- to 2-month intervals when clinical improvement was deemed insufficient, primarily based on the physician’s judgment regarding the ulcer size and pain. While no strict quantitative threshold was used, the decision to proceed with additional sessions beyond three was based on discussion between the patient and physician. If ulcers failed to improve after three TAE sessions, patients were considered for alternative interventions, including surgery. Parameters recorded included radiation dose, fluoroscopy time, and the number of vessels embolized. Statistical analyses were performed using paired t-tests in Excel 365 (Microsoft Corp.).

## Results

### Patients

The patient characteristics and procedural data are summarized in Table 1. The mean duration of symptoms before embolization was 117 months (range, 1–720; median, 12 months). Although one ulcer was of recent onset (1 month), all patients, including this case, had undergone at least 3 months of conservative treatment for chronic symptoms before undergoing embolization. A total of 41 TAE sessions were performed in 17 patients. The mean age was 56.7 ± 16.7 years, and the cohort included seven males and ten females. One patient had Klippel–Trenaunay syndrome, and another had pemphigoid. The mean follow-up was 11.5 ± 3.7 months (range, 6–24 months). Ulcer improvement (defined as a ≥ 50% reduction) was observed in 15 patients (88.2%).

**Table 1:** Patient baseline characteristics and interventional characteristics

Mean age, years (SD)	56.7 ± 16.7
Sex, male/female	7 (41%), 10 (59%)
Laterality, left/right/bilateral	10 / 4 / 3 (59%/24%/18%)
Presence of skin ulcers	17 (100%)
Mean duration of the symptoms, range, median (month)	117 (1–720, 12)
History of deep vein thrombosis	0 (0%)
Preinterventional antiplatelet medication	2 patients (12%)

*SD* Standard deviation

### Procedure and Safety

**Fig. 1:**
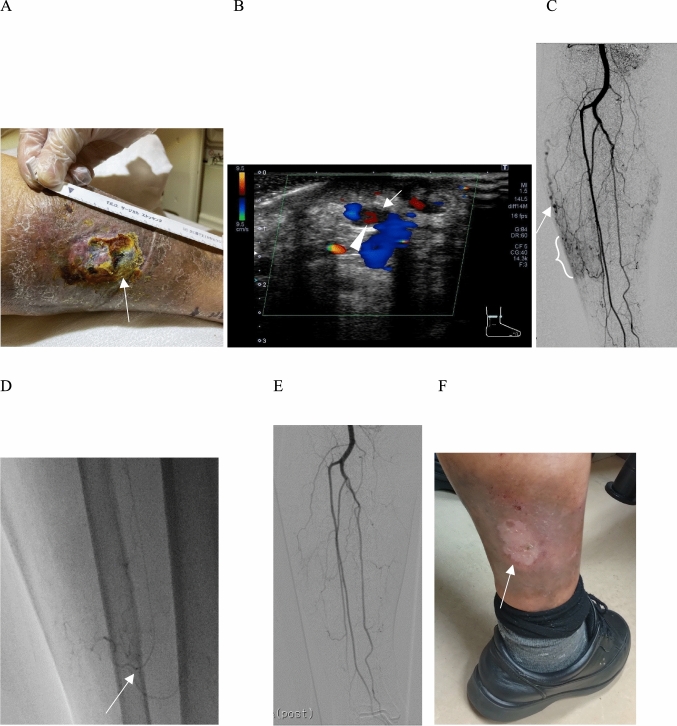
Clinical progression and transcatheter embolization of a lower limb ulcer caused by microarteriovenous fistulas (m-AVFs). **A** The patient experienced a lower limb ulcer and mild pain, which persisted despite prolonged compression therapy performed at another hospital five years previously. The ulcer showed no signs of improvement upon referral to our hospital (the arrow indicates the ulcer site). **B** Ultrasonography with Doppler examination revealed arterial-to-venous blood flow indicative of m-AVFs. The arrow indicates arterial inflow, and the arrowhead indicates a vein. **C** Summed digital subtraction angiography (DSA) demonstrated faint, ill-defined enhancement (white curly brace) and early venous drainage (arrow) originating from the anterior tibial artery, suggesting m-AVFs. **D** Selective catheterization of the anterior tibial recurrent artery was performed using a microcatheter (arrow), followed by embolization with IPM/CS. Mild pain occurred at the embolization site but resolved quickly. **E** Post-embolization DSA showed complete occlusion of the distal anterior tibial recurrent artery, confirming technically successful embolization. **F** At the 2-month follow-up, the ulcer healed completely, leaving residual pigmentation and minimal scarring (arrow)

The procedural data are shown in Table 2. On average, patients received 2.4 ± 1.6 TAE sessions: Six required one session, five required two sessions, two required three sessions, three required four sessions, and one underwent seven sessions. The treated arteries were branches of the anterior tibial artery in 18 procedures, the posterior tibial artery in 28 procedures, the peroneal artery in 13 procedures, the popliteal artery in 9 procedures, and the profunda artery in one procedure. Figure 1 shows a typical case of an m-AVFs. Technical success—elimination of early venous shunting—was 100%. In two procedures, IPM/CS alone did not fully occlude the m-AVFs, necessitating the use of additional gelatin sponge. Mean IPM/CS usage per procedure was 0.41 ± 0.05 g. Clinical improvement after the initial TAE was insufficient in 11 patients, who consequently underwent repeat embolization until adequate ulcer healing or pain relief was achieved.

**Table 2:** Outcome characteristics

DSA findings	
Faint and ill-defined borders enhancements, n (%)	17 (100%)
Early venous return, n (%)	17 (100%)
Number of TAE	41 times
Amount of IPM/CS used for the embolization per procedure (g)	0.41 ± 0.05
Mean radiation dose for the procedure (mGy)	158.4 ± 130.8
Mean fluoroscopy time (minutes)	35.4 ± 14.1
Mean procedural time (minutes)	100.7 ± 42.5
Number of TAE (range and median) of each case	2.4 ± 1.6 (1–7, 2)
Mean number of treated vessels in a single TAE (range and median)	1.7 ± 0.8 (1–4, 2)
Treated vessels, n	69
Branches of the anterior tibial artery, n (%)	18 / 69 (26.1%)
Branches of the posterior tibial artery, n (%)	28 / 69 (40.6%)
Branches of the peroneal artery, n (%)	13 / 69 (18.8%)
Branches of the popliteal artery, n (%)	9 / 69 (13.0%)
Branches of the profunda artery, n (%)	1 / 69 (1.4%)
Adverse events	–
Bleeding complications, n (%)	0 / 41 (0%)
Pain after embolization, n (%)	8 /41 (19.5%)
Mean NRS before the procedure	2.1 ± 1.8
Mean NRS 1 month after the procedure	0.6 ± 1.1
Mortality at 1 month, n (%)	0 (0%)

*IPM/CS* Imipenem/cilastatin sodium, *DSA* Digital subtraction angiography, *TAE* Transarterial embolization, *NRS* Numerical rating scale

Minor adverse events occurred, but none reached grade ≥ 3. Pain at the embolization site appeared in 19.5% of procedures, generally resolving rapidly. One patient had mild, persistent pain for a week, and three had brief rashes or itching. No hematomas, infections, distal ischemia, or pulmonary emboli were documented.

### Ulcer Outcomes

Six patients achieved healing after a single session. Among the remaining 11, one experienced recurring ulcers requiring four sessions, and another relapsed at three months. One complex case underwent seven sessions, resulting in only partial improvement; subsequent imaging revealed pelvic vein stenosis and lymphatic dysfunction.

### Pain Outcomes

Pain scores were assessed at one month after the final TAE session. One patient reported mild, persistent pain during the first postoperative week, which resolved spontaneously by the 1-month follow-up. The mean NRS score declined from 2.1 ± 1.8 before TAE to 0.6 ± 1.1 after treatment (*p* < 0.05). All 17 patients were therefore considered to have achieved pain relief.

## Discussion

TAE with IPM/CS proved safe and effective in treating m-AVFs of the lower limb, offering ulcer healing and pain relief. These results partially align with earlier findings by Okuno et al., which demonstrated that IPM/CS embolization alleviates pain in musculoskeletal disorders by targeting inflammation-related neovessels [12]. However, in m-AVF-related skin ulcers, the underlying mechanism appears to be hemodynamic; specifically, pathological microvascular shunting leads to venous hypertension and tissue congestion. Thus, while both conditions may involve abnormal neovascularization, the therapeutic targets differ fundamentally. In addition to venous congestion, arteriovenous shunting may also cause a steal phenomenon, diverting arterial blood from surrounding tissues, and lowering skin perfusion pressure, which could contribute to ulcer persistence. In m-AVFs, the goal of embolization is to interrupt abnormal arteriovenous connections and restore physiological perfusion. We also observed early improvements in pain and ulceration following embolization. Our 100% technical success rate suggests that IPM/CS particles can occlude multiple small-caliber arteries.

Although embolization procedures are often associated with a risk of ischemia and potential ulcer deterioration, IPM/CS differs fundamentally from conventional permanent embolic agents such as polyvinyl alcohol particles. When mixed with contrast, IPM/CS forms poorly soluble particles that dissolve relatively quickly in vivo, resulting in only a transient embolic effect. This transient occlusion selectively reduces pathological shunting while allowing timely restoration of normal tissue perfusion, thereby minimizing the risk of skin necrosis or ulcer aggravation. In our cohort, no cases of ulcer worsening were observed after embolization, supporting the favorable safety profile of IPM/CS for treating ulcerative lesions. The therapeutic effects of IPM/CS in this context are presumed to arise from its selective, transient occlusion of abnormal microvasculature, which relieves venous hypertension and tissue congestion. Given the small quantity of IPM/CS delivered intra-arterially, any contribution of its antibiotic properties is unlikely to be significant.

However, technical success—as defined by the angiographic elimination of early venous shunting—did not always correspond to immediate or sustained clinical improvement, as 11 patients required additional embolization. One patient was later found to have pelvic vein stenosis and lymphatic dysfunction. Patients with deep venous or lymphatic abnormalities were excluded from this study based on preprocedural imaging and clinical evaluation; thus, these factors are unlikely to have contributed to treatment failure. Other potential reasons include premature dissolution of IPM/CS, residual microshunting below angiographic resolution, or the possibility that m-AVFs were not the primary cause of ulceration in some instances. These findings highlight that angiographic endpoints alone are insufficient to predict clinical success and emphasize the importance of vigilant follow-up [7]. Causes include peripheral artery disease, diabetes, pressure ulcers, rheumatic diseases, collagen diseases, skin disorders, and infections, all of which ultimately result in compromised tissue perfusion, necrosis, and ulceration [5, 16–21]. Although m-AVFs represent a less recognized cause of lower limb ulcers, our findings support the hypothesis that increased tissue pressure due to m-AVFs contributes to the development of refractory skin ulcers. A proper understanding of m-AVF-related ulcers requires recognition of venous congestion as a key underlying mechanism [7]. Currently, there is no standardized treatment strategy for m-AVFs, and surgical intervention remains challenging because numerous small fistulas can be complex to identify and occlude. Additionally, sclerotherapy has limited efficacy for fast-flow lesions such as m-AVFs [9].

### Limitations

This study has some limitations. First, the small sample size and retrospective design limit the generalizability of our findings. Second, although most patients were treated using a single embolic agent (IPM/CS), gelatin sponge was used as an adjunct in two procedures when complete occlusion could not be achieved. As a result, the therapeutic effects observed in this study cannot be attributed solely to IPM/CS. Third, baseline pain levels in this cohort were relatively mild, which may have underestimated the potential analgesic effects of the intervention. Although no skin necrosis was observed in our cohort, embolization of multiple arterial branches carries an inherent risk of distal ischemia and may, in rare cases, lead to skin ulceration. Clinicians should exercise caution to minimize the number of arteries embolized and ensure that perfusion to the surrounding tissues is preserved.

## Conclusion

TAE with IPM/CS for m-AVFs causing refractory skin ulcers of the lower limbs is a feasible and safe treatment option. However, these findings are based on a small, retrospective cohort, and further prospective studies are needed to validate the effectiveness and safety of this approach.
